# Cognitive behavioural treatment for mild Alzheimer’s patients and their caregivers (CBTAC): study protocol for a randomized controlled trial

**DOI:** 10.1186/s13063-015-1043-0

**Published:** 2015-11-17

**Authors:** Simon Forstmeier, Andreas Maercker, Egemen Savaskan, Tanja Roth

**Affiliations:** Developmental Psychology, Faculty II, University of Siegen, Adolf-Reichwein-Str. 2, 57068 Siegen, Germany; Psychopathology and Clinical Interventions, Department of Psychology, University of Zurich, Binzmuehlestrasse 14/17, 8050 Zurich, Switzerland; Clinic for Geriatric Medicine, Psychiatric University Hospital, University of Zurich, Minervastrasse 145, 8032 Zurich, Switzerland

**Keywords:** Alzheimer’s, anxiety, apathy, CBT, cognitive behavioural therapy, dementia, depression, neuropsychiatric symptoms, psychosocial intervention, randomized controlled trial

## Abstract

**Background:**

About 90 % of all persons with mild Alzheimer’s disease experience neuropsychiatric symptoms, most frequently apathy, depression, anxiety and irritability. These symptoms are associated with greater morbidity, a reduced quality of life for the patient, an increased burden and depression for the caregiver, and higher costs of care and nursing home placement. Psychosocial interventions based on behaviour therapy represent the most efficacious treatment of neuropsychiatric symptoms. However, there is no study, to our knowledge, that has evaluated a multicomponent treatment programme based on comprehensive, cognitive behavioural therapy (CBT). This randomized controlled trial aims to evaluate a CBT-based treatment programme consisting of 8 modules and 25 sessions.

**Methods/design:**

Fifty patients with mild Alzheimer’s disease alone or with mild mixed dementia (Alzheimer’s disease and vascular dementia) who have any neuropsychiatric symptom will be included. A caregiver must be available. The patients and their caregivers will be randomized to either the CBT-based intervention group or to the control condition group, which receives treatment as usual. The primary outcome measure is depression in the patient with Alzheimer’s disease. The secondary outcome measures for a person with Alzheimer’s disease are other neuropsychiatric symptoms, quality of life and coping strategies. The secondary outcome measures for a caregiver are caregiver’s burden, depression, anxiety, anger, quality of life and coping strategies. Neuropsychological testing includes tests of cognitive function and activities of daily living and a global clinical assessment of severity. Participants in both groups will be assessed before and after the treatment phase (lasting approximately 9 months). Follow-up assessments will take place 6 and 12 months after treatment. All assessments will be conducted by blinded assessors.

**Discussion:**

This trial has the potential to establish an empirically based psychological treatment for non-cognitive symptoms that reduce the quality of life of a person with dementia and a caregiver. This treatment approach focuses not only on the person with dementia, but also on the caregiver and on the dyad. The treatment manual will be published and training workshops will be offered, so that the information can be widely spread among healthcare professionals.

**Trial registration:**

ClinicalTrials.gov NCT01273272.

## Background

Alzheimer’s disease is a chronic debilitating mental condition manifested by cognitive and memory deterioration, progressive impairment in performing the activities of daily living and a variety of neuropsychiatric symptoms and behavioural disturbances. In 2006, the worldwide prevalence of Alzheimer’s disease was 26.6 million; this number is predicted to quadruple by 2050 [1]. Mild cases of Alzheimer’s disease make up the largest proportion of all Alzheimer’s disease cases, with 15 million worldwide.

### The medical problem

Neuropsychiatric symptoms are very common among patients with Alzheimer’s disease [2]. Affective and behavioural symptoms may be present in early dementia or emerge later in the course of the illness. The most frequent disturbances in mild Alzheimer’s disease are apathy, depression, anxiety and irritability, with a total prevalence estimated to be up to 92 % [3]. Almost all patients with Alzheimer’s disease are affected by neuropsychiatric symptoms at some point. Even with mild cognitive impairment, a condition characterized by cognitive deficits without an impairment in functional activities, 35–73 % of patients experience neuropsychiatric symptoms [4]. Neuropsychiatric symptoms are associated with greater morbidity and a reduced quality of life for the patient, as well as an increased burden and depression for the caregiver [5] and higher costs of care [6]. They are also associated with the increased use of psychotropic medications and increased patient and caregiver abuse [7]. Neuropsychiatric symptoms are a primary predictor of nursing home placement [8]. Thus, interventions aimed at treating these symptoms might have a tremendous effect on patients, caregivers and society.

### Evidence for psychosocial interventions

A whole variety of different approaches can be described as psychosocial (or non-pharmacological) interventions for dementia patients [9]. However, empirical evidence is lacking for many of the approaches, as some reviews have concluded [9–11]. The proposed study is aimed at meaningfully combining interventions whose effectiveness is empirically supported. In their wide-ranging and comprehensive review, Livingston et al. [10] rated a number of approaches by the level of evidence and graded their recommendation of each approach, based on the Oxford Centre for Evidence-Based Medicine criteria. The only approaches that received the highest grade of recommendation were behavioural management techniques focused on the caregiver, including psychoeducation and interaction training. At second position were behavioural management techniques focused on the person with dementia, including techniques focusing on pleasant activities and problem solving, multisensory stimulation, active music therapy and cognitive stimulation. The guidelines published by the UK National Collaborating Centre for Mental Health [11] concluded that, based on the evidence, behavioural management was most effective in reducing a challenging behaviour, while cognitive behavioural therapy (CBT) was most effective in reducing depression and anxiety in people with dementia. Thus, a psychosocial intervention based on (cognitive) behaviour therapy seems to be one of the most promising treatments of non-cognitive symptoms in patients with Alzheimer’s disease. Next, we summarize evidence for concrete cognitive behavioural and related interventions, which are easily integrated into a CBT approach.

#### Engagement in pleasant or structured activities

Teri et al. [12] reported empirical evidence from a controlled clinical trial of two CBT-based interventions to treat depression in people with Alzheimer’s disease. One treatment involved increasing engagement in pleasant activities and events and the other involved behavioural problem-solving strategies. The participants in both intervention groups showed significant improvements in depressive symptoms compared with those in the two control groups, and these improvements were maintained for 6 months after the trial. The ‘pleasant events’ treatment resulted in a large effect size (*d* = 0.9–1.7 for three different depression measures), with 52 % clinically significant improvement (versus 20 % in the control groups). In addition, there were also significant improvements in caregivers’ depression scores, but no improvement for the caregivers in the control groups. Increasing engagement in pleasant activities, including social, physical and leisure activities, is not only important for reducing depression, but also decreases the amount of daytime sleep taken, improves night-time sleep [13] and reduce wandering, aggression and agitation [14].

#### Training caregivers in behaviour management techniques

In a randomized controlled trial, exercise and behaviour management techniques led to significant improvements in depression and physical health, e.g., behavioural intervention reduced symptoms of depression more than twice as much as routine medical care [15]. Behaviour management techniques are also effective in improving food intake [16], reducing urinary incontinence [17] and improving functional abilities, such as dressing [18].

#### Interventions for the caregiver

Caring for a patient with Alzheimer’s disease is associated with a severe negative impact on the caregiver’s health. In caregivers of patients with Alzheimer’s disease, the prevalence of depressive disorders is 22 % [19] and that of anxiety is 25 % [20]. Therefore, much research has focused on psychosocial interventions for caregivers of patients with dementia [21, 22]. Interventions to improve the health status of caregivers also have positive effects on neuropsychiatric symptoms of patients with Alzheimer’s disease. Therefore, a comprehensive treatment approach with a focus on patients’ mental health should also include interventions to reduce the burden on their caregivers. The treatment effect was found to be higher when the intervention included CBT-based psychotherapy [23], the number of sessions was more than ten, both caregiver and patient were involved and the treatment was individualized [21]. Effect sizes of CBT interventions were on average medium (*d* = 0.68) [21] or large (*d* = 1.20) [23] with regard to mood measures (depression, anxiety).

#### Cognitive restructuring

As well as behavioural techniques, cognitive restructuring is one of the major techniques in CBT. In their cognitive model of depression, Beck et al. [24] describe cognitive schemas as core components of emotional disorders, e.g., a negative view of oneself, the world and the future (the ‘cognitive triad’) in the case of depression. Cognitive behavioural therapy includes strategies to change those negative thoughts and beliefs. Since the individual’s ability for introspection and reflection is a prerequisite of cognitive restructuring, and this ability is progressively impaired in dementia, it has been suggested that this technique is most likely to benefit patients with mild dementia [25]. The point beyond which cognitive restructuring is not useful anymore has yet to be identified. There has been no randomized controlled trial yet, but case studies recommend the potential use of cognitive techniques for patients with mild Alzheimer’s disease [26, 27]. Clearly, some modifications are necessary, e.g. simplifying the material, enhancing encoding by having the patient repeat information, using reminder cards with helpful thoughts and involving caregivers to facilitate learning at home.

#### Structured life review

A structured life review was shown to be effective in treating depression in older people [28, 29]; in fact, it is among the most effective treatments for depression in older age [30]. A structured life review involves individual sessions, in which a person is guided chronologically through life experiences and is encouraged to evaluate them [31, 32]. It is more effective than unstructured reminiscence therapy, one of the most popular psychosocial interventions in dementia (effect size: 0.92 versus 0.46, respectively) [28]. A life review is increasingly used for people with dementia [33] and is effective in reducing their depression [34, 35]. Effect size is in average small for cognition (*g* = 0.33) and depression (*g* = 0.31) [29]. It helps patients with Alzheimer’s disease gain access to past memories and thereby enhances their sense of personal identity, maintains their self-worth and offers them a pleasurable experience.

#### Couples counselling

All previous psychotherapeutic strategies have focused either on the patient with Alzheimer’s disease or the caregiver. Recent research tries to treat a couple facing Alzheimer’s disease as a unit that needs to be the focus of the treatment. To be a partner of the other is one of the greatest resources for both the patient and the spouse, and psychotherapy should aim at improving the marital relationship, communication and the attitude to face the future more optimistically and collaboratively. The typical topics covered in couples counselling are: expressing and discussing fears openly; gradually challenging the view that everything is continuing normally; adapting to new roles (dependence, responsibility); improving communication style; establishing joint activities; identifying old and new coping strategies; and maintaining important elements of the former relationship despite the changes necessitated by the illness [36]. A further frequent topic is the joint planning of future care, with a discussion of uncertainties and worries experienced by the patient and the caregiver [37]. The first results of an intervention study of couples counselling are very encouraging [38]; however, there were no results from randomized controlled trials at the time of writing.

While there is good evidence for most of the aforementioned psychotherapeutic approaches, there have been only a few attempts to evaluate a comprehensive CBT programme including (almost) all of the described strategies. We outlined our comprehensive CBT programme, as described next, in a German textbook [39]. Several case studies have been described, which include engagement in pleasant activities, behaviour management techniques and cognitive restructuring, but not life review, couples counselling, and interventions for the caregiver [40, 41]. A randomized controlled trial with a brief CBT-based intervention, including structured activities and a life review, has recently been conducted, with mixed results (a significant reduction only in female participants) [42]. Furthermore, self-maintenance therapy, a short-term residential treatment programme with CBT elements, a life review and interventions for the caregiver resulted in a significant decrease in depression and problematic behaviour compared with the baseline [43].

Not only are efficacy data missing for a comprehensive, multicomponent, CBT-based treatment programme, but most previous studies exhibited methodological weaknesses, such as the lack of an adequate control condition, outcome measures restricted in number and domain and involvement of either the patient or the caregiver but not both.

### Rationale for the current study

The current study combines theory-based, multicomponent intervention strategies, whose efficacy has been demonstrated in randomized controlled trials or supported by case studies, with state-of-the-art trial methodology. The study tries to overcome the shortcomings of previous research in several respects: (1) the interventions are based on established psychological models; (2) CBT strategies will be consistently applied; (3) a high quality of treatment and consistency in treatment delivery will be ensured by using trained psychotherapists with specialized training in the intervention programme and regular supervision during the study; (4) the targets of the interventions will be the patient with Alzheimer’s disease, the caregiver and the dyad; (5) the treatment will be structured, but also individualized according to individual problems; (6) a randomized, controlled, assessor-blinded study design will be employed; (7) various outcome measures, involving various domains of the patient’s and caregiver’s health, will be applied; (8) statistical power calculations will be used to estimate the sample size.

A modular and non-uniform programme is justified because no single strategy would achieve the treatment goal to the desired extent. Modular programmes are also common for other mental disorders, e.g. dialectical behavioural therapy for treating borderline personality disorder or CBT for treating depression.

The trial manual includes eight modules. The content of the sessions is described in an unpublished manual. Because the patient and the caregiver will have different symptomatologies (e.g., depression, apathy, anxiety and irritability) and varying degrees of marital problem, certain modules may be more important than others. Therefore, the therapist can assign different weights to the eight modules, while still conforming to the study protocol. The modules are as follows:

1. *Diagnosis and goal setting* This first module includes a thorough diagnosis of emotional and behavioural problems of the patient and caregiver, a behaviour analysis of the situation, including behaviours, emotions, cognition and consequences and individual goal setting. A therapeutic alliance will be developed during this phase and motivation for change is encouraged by the therapist [44].
2. *Psychoeducation* Sometimes, but not always, patients and caregivers are inadequately informed of the diagnosis of Alzheimer’s disease, its causes, course and treatment options. If this is the case, the therapist provides appropriate information. Psychoeducation is not efficacious in reducing symptoms as a stand-alone intervention [10], but is a motivational prerequisite for change. Part of psychoeducation is information and training on adopting external memory aids. This is not an efficacious intervention on its own either [45], but these strategies are needed to support subsequent intervention and related therapeutic tasks.
3. *Engagement in pleasant activities* Based on the behavioural theory of depression [46], the patient is helped to increase the number and frequency of pleasant activities engaged in and to adopt a structured weekly schedule. Social, physical and other leisure activities are included.
4. *Cognitive restructuring* Based on the cognitive model of emotional disorders [24], dysfunctional thoughts (depression, anxiety or anger related) will be recorded and discussed and alternative, helpful thoughts will be practised.
5. *Life review* The structured life review that we adopt in our intervention programme [31, 32] is based on Erik Erikson’s eight stages of man [47]. In each life review session, one or two of Erikson’s stages are applied to the life of the patient. The patient is guided chronologically through life experiences and is encouraged to evaluate them. Photographs, music and other artefacts are used to support reminiscence.
6. *Training caregiver in behaviour management techniques* The system model of behaviour regulation [44] describes each problem behaviour as triggered by precursors and followed by consequences (similar to the ‘ABC of behaviour change’ outlined by Teri *et al*. [48, 15]). Strategies to change precursors and consequences are planned, and the caregiver is trained in applying them.
7. *Interventions for the caregiver* Interventions with the aim of improving the caregiver’s well-being, stress management and emotional regulation (e.g. anger management) and social support are mainly delivered to the caregiver in sessions without the patient and in parallel with patient sessions. These treatment strategies are based on the cognitive model of emotional disorders [24], the system model of behaviour regulation [44] and the buffering theory of social support [49]. Interventions from modules 3 and 4 are used; however, the focus of this module is on the caregiver and not on the patient or the dyad (module 8).
8. *Couples counselling* The vulnerability–stress–adaptation model of marriage [50] assumes that vulnerability factors (e.g. attachment style) and stressful events (e.g. dementia) impact the adaptive competencies of a couple. The adaptive processes (e.g. communication, joint coping) influence, in turn, the stressor and marital satisfaction. Therefore, a few sessions, which are focused on expressing fears, adapting to new roles, improving communication and joint coping, establishing joint activities and planning for the future, are included. To help the couple to talk about the relationship and develop a positive attitude towards couples counselling, an oral history interview is used [51].

### Study objectives

The goal of this study is to evaluate the effect of a CBT-based, multicomponent treatment programme on the health of patients with mild Alzheimer’s disease and their caregivers. The main focus is to improve the patients’ health, but the inclusion of the caregivers is necessary because there is a reciprocal interaction between the well-being of patients and their caregivers. We expect, in comparison with the control condition, a significant decrease in depressive and other neuropsychiatric symptoms and an improvement in functional abilities, quality of life and adaptive coping strategies, including social support, for the patient. Also expected are a decrease in depressive, anxiety and anger symptoms and the burden of providing care and improvements in the quality of life and adaptive coping strategies, including social support, for the caregiver.

## Methods/design

### Design

This is a randomized, controlled, assessor-blinded, follow-up study, which includes patients with mild Alzheimer’s disease and their caregivers. The patients and their caregivers will be randomized to either the CBT-based intervention group or to the control condition group, which receives treatment as usual.

#### Intervention condition (cognitive behavioural therapy)

The CBT-based, multicomponent treatment programme consists of 25 weekly or biweekly sessions. It includes the following eight modules: diagnosis and goal setting; psychoeducation; engagement in pleasant activities; cognitive restructuring; life review; training the caregiver in behaviour management techniques; interventions for the caregiver; and couples counselling. While the treatment is conducted according to the manual, it is to be individualized according to individual problems of the patient and the caregiver. The interventions are described next in more detail.

#### Control condition (treatment as usual)

The patients in the control condition group receive standard medical and psychosocial care, which is treatment as usual at the Psychiatric University Hospital Zurich. We have operationalized the treatment-as-usual condition in such a way that it is partly manualized, but still individualized. Each patient or caregiver must receive at least three out of the six interventions: (1) psychoeducation on dementia and its treatment (oral and written); (2) appropriate medical treatment; (3) social counselling by specialized staff; (4) memory training in a group setting; (5) a self-help group for the patient; and (6) a self-help group for the caregiver. Thus, patients receive usual care with minimal support, which is ethically acceptable, since the standard treatment is implemented in the control condition. At the same time, using this control condition is methodologically appropriate because the likelihood of a significant effect is expected to be smaller than that in the intervention condition. Moreover, the recruitment of study subjects will not be compromised by using this treatment-as-usual control condition. Members of the staff of the Psychiatric University Hospital Zurich, who are independent of the CBT interventionists, will provide the control intervention.

**Fig. 1:**
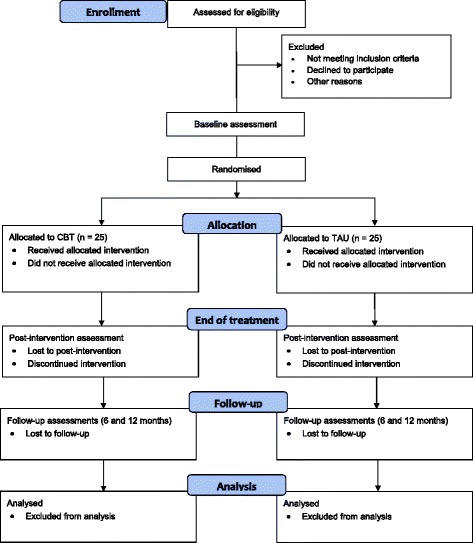
Trial design and CONSORT flow diagram. CBT, cognitive behavioural therapy; TAU, treatment as usual

Enrolment into one of the two conditions will be done consecutively during the recruitment phase. The treatment phase takes an estimated average of 9 months (including 25 weekly or biweekly sessions in the CBT condition). The participants in both conditions will be assessed before and after the treatment phase. Follow-up assessments will take place 6 and 12 months after treatment. All assessments will be conducted by blinded assessors. A CONSORT-style flow chart for the trial is shown in Fig. 1.

### Participants

#### Recruitment

The participants will be recruited from three sources: (1) the Psychiatric University Hospital Zurich, Department of Gerontopsychiatry; (2) the outpatient clinic of the University of Zurich, Department of Psychopathology and Clinical Intervention; and (3) other geriatric or memory clinics and general practitioners in the greater area of Zurich. Patients are referred to our two clinics either for confirmation of possible dementia at the memory clinic of the Psychiatric University Hospital Zurich or for treatment of affective symptoms. Since mild cases of Alzheimer’s disease are often treated by a general practitioner until the symptoms progress to a moderate stage, the network of general practitioners in Zurich complements recruitment at our two clinics and other clinics. Patients recruited via a general practitioner will receive a thorough neuropsychological and medical assessment at the Psychiatric University Hospital Zurich.

#### Inclusion and exclusion criteria

Patients must meet the criteria of the National Institute of Neurological and Communicative Disorders and Stroke – Alzheimer’s Disease and Related Disorders Association (NINCDS–ADRDA) for probable or possible Alzheimer’s disease [52]. Mixed Alzheimer’s and vascular dementia cases will also be included. Only cases of Alzheimer’s disease with a mild severity of dementia will be included, as determined by the Clinical Dementia Rating Scale (i.e., scores of 0.5 or 1) [53] and by the Mini Mental State Examination (i.e., scores of 20 or more) [54]. The patient must experience any non-cognitive symptom that motivates the acceptance of psychotherapeutic help. Typically, symptoms of depression, apathy, anxiety or irritability are present at the time of enrolment. A caregiver must be available to take part in most of the psychotherapy sessions. This is typically the partner, but a child or a good friend, who is able to take responsibility for reminding the patient about therapeutic tasks, is also accepted.

Exclusion criteria are a concomitant alcohol or drug addiction and a history of a malignant disease, severe organ failure, metabolic or haematological disorders, neurosurgery or a neurological condition, such as Parkinson’s disease, epilepsy, postencephalitic or postconcussional syndrome.

#### Informed consent

The objectives and goals and detailed information about the assessment and therapy, as well as the procedure of randomization, will be explained to the subjects. The information will also be given in printed form to the subjects. Written informed consent will be obtained from all participants and caregivers prior to inclusion. Patients who cannot give their informed consent will not be included in the study.

#### Withdrawal criteria

Participation in the study is voluntary. There are no negative consequences for non-participation or dropping out. Patients who deny participation or drop out will receive medical and psychosocial care as usual.

#### Sample size

Previous studies of the interventions for patients with mild Alzheimer’s disease included in our multicomponent treatment programme found small-to-medium effect sizes for depression outcomes (*d* = 0.4 to 0.6, corresponding to *f* = 0.2 to 0.3) [12, 15, 21, 43]. We base our sample size calculation on the assumption of a small-to-medium effect size for depression as measured using the Geriatric Depression Scale (*d* = 0.5, *f* = 0.25). The sample size was calculated using G*Power 3.1.0 software [55]. Assuming *α* = 0.05 and a test power 1 − β = 0.80, a total sample size of *n* = 44 is required to test condition × time interactions in an analysis of variance (ANOVA) with repeated measurements (*r* = 0.3). Since previous studies on CBT-based psychotherapy for caregivers of people with dementia found medium [21] or large [23] effect sizes on mood measures (depression, anxiety), this sample size should also be sufficient to find an effect of the intervention on caregiver’s mood. We assume a rather conservative rate of loss of 60 % from the screening of eligibility to the enrolment of subjects. Thus, we propose to start with approximately 120 patients and caregivers at the screening, which we expect to reduce to 50 at enrolment. A drop-out rate of approximately 10 % would lead to *n* = 44.

### Randomization

After providing informed consent and receiving a baseline assessment, the participating couples will be individually randomized. The allocation ratio for randomization into either the intervention or control condition is 1:1. The randomization will be performed by a computer algorithm independently operated by the Clinical Trial Centre of the University Hospital Zurich. The results of the randomization will be sent to the study nurse, who will telephone the participant and the caregiver to explain the next steps.

### Cognitive behavioural therapy intervention

#### Session content and setting

The CBT-based, multicomponent treatment programme consists of 25 sessions, mostly 60 mins in duration. The first two sessions (diagnosis and behaviour analysis) may take up to 90 mins. Table 1 details the content of all sessions. Most of them are joint sessions, but some are separate sessions with either the patient or the caregiver. The sessions of module 7 (interventions for the caregiver) are not prescribed by number, but as many are delivered as needed. They are conducted in parallel with sessions 4 to 16, with a variable time between the sessions starting after session 3. All sessions occur in rooms of the Psychiatric University Hospital Zurich, Department of Gerontopsychiatry, or at the outpatient clinic of the University of Zurich, Department of Psychopathology and Clinical Intervention.

**Table 1:** Description of sessions

No	Topic	Therapeutic strategies	Setting
Module 1: Diagnosis and goal setting			
1	Diagnostic investigation	Diagnostic interview: comprehensive assessment of affective and behavioural symptoms, burden of caregiver, resources	Joint
2	Behaviour analysis and goal setting	Analysis of situation, behaviours, emotion, cognition, consequences; setting individual goals; planning interventions for joint and separate sessions	Joint
Module 2: Psychoeducation			
3	Psychoeducation	Providing information regarding disease, course, cause, medication, psychosocial interventions, support possibilities; adopting external memory aids (notebook, lists, signs for orientation, etc.); introducing mood journal	Joint
Module 3: Engagement in pleasant activities			
4	Setting the stage for pleasant activities	Explaining relation of behaviour (e.g., inactivity), mood (e.g., depression), neuronal degeneration and cognitive decline; developing a personal list of pleasant activities; introducing activity journal	Joint
5	Planning of pleasant activities	Selection of pleasant (social, physical, leisure) activities; planning of pleasant activities in a structured weekly schedule	Possibly without caregiver
6	Establishing regular activities	Discussing problems and progress with regular pleasant activities; motivation strategies to form habits (reinforcing by rewards; positive self-talk); introducing thought journal	Joint
Module 4: Cognitive restructuring			
7	Setting the stage for cognitive restructuring	Assessing typical negative (depression, anxiety, or anger related) and positive thoughts; completing the event–thought–emotion schema on the basis of recent examples; finding alternative thoughts	Joint
8	Challenging negative thoughts	Repetition of event–thought–emotion schema; challenging dysfunctional thoughts; repetition of finding alternative thoughts; describing thought control techniques (card and signal techniques)	Possibly without caregiver
9	Practising helpful thoughts	Discussing problems and progress with thought control techniques; strategies for practising helpful thoughts; introducing life review: structure and tools (e.g., photographs)	Joint
Module 5: Life review			
10	Childhood	Reminiscence of positive experiences in childhood	1 or 2 sessions joint, 2 or 3 sessions without caregiver
11	Adolescence	Reminiscence of positive experiences in adolescence	
12	Young adulthood	Reminiscence of positive experiences in young adulthood	
13	Older adulthood	Reminiscence of positive experiences in older adulthood; integration: review significant successes, reframe difficult times	
Module 6: Training caregiver in behaviour management techniques			
14	Setting the stage for behaviour management	Identifying problem behaviours, their precursors and consequences; describing the techniques: planning and implementing an intervention; evaluating an intervention; providing the caregiver (and patient) with methods to change precursors and consequences of behaviour (part 1)	Joint
15	Changing problem behaviour, part 1	Discussing the methods to change precursors and consequences of behaviour (part 1); discussing progress; discussing methods to change precursors and consequences of behaviour (part 2)	Joint
16	Changing problem behaviour, parts 2 and 3	Discussing progress; discussing the methods to change precursors and consequences of behaviour (part 3) (or repetition of behaviour parts 1 and 2)	Possibly without patient
Module 7: Interventions for the caregiver (parallel to sessions 4–16)			
i	Stress management and emotion regulation	Analyzing stressors, thoughts, emotional and behavioural reactions; self-monitoring of thought and behaviour; training in problem solving, cognitive reframing, relaxation techniques	Only caregiver
ii	Pleasant activities	Developing a personal list of pleasant activities; keeping an activity journal; selection of pleasant (social, physical, leisure) activities; planning of pleasant activities in a structured weekly schedule; discussing problems and progress with regular pleasant activities	Only caregiver
iii	Social support	Analyzing social support network; acceptance of formal and informal support (including cognitive restructuring); strategies of utilization of social support; communication with others about burden	Only caregiver
iv…		Approximately four sessions, but as many as needed	Only caregiver
Module 8: Couples counselling			
17	Setting the stage for couples counselling	Analyzing emotions regarding relationship, wishes, expectations, fears, typical conflicts, etc.; identifying core problems; oral history interview	Joint
18	Communication and joint problem-solving training	Improving communication style (roles as speaker and listener), training communication sequences; identify old and new coping strategies; establishing joint activities; maintaining important elements	Joint
19	Acceptance and planning for the future	Gradually challenging the view that everything is continuing normally; adapting to new roles (dependence, responsibility); joint planning of future care with a discussion of uncertainties and worries experienced by patient and caregiver	Joint
Closing of therapy			
20	Summary and reflection	Goal attainment scaling; evaluation of strategies; future planning	Joint

#### Therapeutic techniques

The majority of the therapeutic techniques stem from the CBT literature, including behaviour analysis, psychoeducation, advice on establishing pleasant activities, guidance on self-reward to motivate oneself, cognitive restructuring through Socratic dialogue and guided discovery, training in behaviour management, problem solving and stress management. The life review, though not a CBT technique in origin, is used in a highly structured variant to fit into the general CBT approach. The techniques in the couples counselling sessions, which do not belong to traditional CBT either, mainly include adopted behavioural strategies, i.e., training in communication and problem solving, planning and cognitive restructuring.

#### Psychotherapists

The CBT treatment programme will be administered by trained psychotherapists with a master’s degree in psychology or medicine and a qualification in CBT. Furthermore, all participating psychotherapists will be trained for this novel intervention by a panel of experts and supervisors in the field of CBT for older age (SF and AM). A 2-day workshop will precede the intervention phase. Supervision sessions will take part every month (i.e., about five sessions per patient). All sessions will be documented in a structured form and reflected upon in the supervision sessions.

#### Standardization of procedures

The therapeutic strategies and material for each session will be available in written form to all participating psychotherapists. Adherence to the treatment manual is ensured by the intensive training before the intervention phase, by regular supervision and by controlling the session documentation.

### Outcome measures

#### Primary outcome measures for a person with Alzheimer’s disease

The primary outcome measure will be level of depression in the patient with Alzheimer’s disease. Depressive symptoms will be measured using a short form of the Geriatric Depression Scale [56] and the Cornell Scale for Depression in Dementia [57]. Depressive disorders will be assessed using the Structured Clinical Interview for DSM-IV Axis I Disorders [58]. The diagnosis of depression is based on the criteria for major and minor depression of the Diagnostic and Statistical Manual of Mental Disorders IV (Text Revision) [59] and on the provisional diagnostic criteria for depression in Alzheimer’s disease [60]. Both the Cornell Scale for Depression in Dementia and the Geriatric Depression Scale are widely used; while the Geriatric Depression Scale is most appropriate for pre-clinical and mild dementia, the Cornell Scale for Depression in Dementia can also be applied when the severity of dementia is increasing.

#### Secondary outcome measures for a person with Alzheimer’s disease

Other emotional and behavioural symptoms will be measured using the Neuropsychiatric Inventory [61] and the Apathy Evaluation Scale [62]. The Neuropsychiatric Inventory is a structured interview with a caregiver, addressing 12 behavioural and affective domains common in dementia, agitation, irritability, anxiety, dysphoria, hallucinations, delusions, apathy, euphoria, disinhibition, aberrant motor behaviour, night-time disturbances and appetite and eating abnormalities. The Apathy Evaluation Scale is an assessment scale for apathy, and has been validated for patients with Alzheimer’s disease and other dementias.

The quality of life is measured by the Quality of Life (Alzheimer’s Disease) Instrument [63]. It is based on direct interviews with patients with Alzheimer’s disease and a questionnaire completed by caregivers. The advantage of the Quality of Life (Alzheimer’s Disease) Instrument over other scales is that the caregiver version can also be applied to severe stages of Alzheimer’s disease.

The preferred coping strategies for the patient are assessed using the Stress Coping Inventory [64]. It includes 20 subscales, which allow differentiation between strategies targeting stress reduction (‘positive strategies’, e.g., social support, positive self-instruction) and stress increase (‘negative strategies’, e.g., avoidance, resignation).

#### Secondary outcome measures for a caregiver

The caregiver’s burden, i.e., the psychological, psychosocial, physical and financial burden of providing care for a patient with Alzheimer’s disease, is measured using the Zarit Burden Interview [65] and the following well-being and quality of life measures: the Centre for Epidemiologic Studies Depression Scale [66], the trait scale of the State Trait Anxiety Inventory [67], the anger-in and anger-out scales of the State Trait Anger Expression Inventory [68], the Satisfaction With Life Scale [69], the 12-item Short-Form Health Survey [70] and the Stress Coping Inventory [64].

### Neuropsychological assessment and diagnostic procedures

The patient’s cognitive function is primarily tested with the Consortium to Establish a Registry for Alzheimer’s Disease (CERAD) Neuropsychological Assessment Battery [71]. Further cognitive tests are added, to ensure at least two tests per domain. This battery includes:

- *Screening* The Mini Mental State Examination [54];
- *Memory* The CERAD Word List Memory Test (learning, recall and recognition) [72]; a German version of the Auditory Verbal Learning Test [73]; the Corsi Block Task (forward and backward series) [74];
- *Language* The CERAD Animal Naming Task [75]; a modified Boston Naming Test [76]; the Controlled Oral Word Association Test [77];
- *Praxis* The CERAD Constructional Praxis [78]; the Rey–Osterrieth Complex Figure Test [79]; the Picture Completion subtest of the Wechsler Adult Intelligence Scale-III [80];
- *Executive function* Task switching: the Trail Making Test, Part B [81]; Inhibition of prepotent responses: the Stroop Color-Word Test [82]; Updating working memory: Digit Span Backward from the Wechsler Adult Intelligence Scale-III [80];
- *Attention* The Trail Making Test, Part A [81]; the Digit Symbol Substitution Test from the Wechsler Adult Intelligence Scale-III [80].

Activities of daily living will be assessed using the Barthel Index [83], which is the standard measure in German-speaking countries [84]. Instrumental activities of daily living will be assessed using the Bayer Activities of Daily Living Scale [85], which is a 25-item, informant-rated, internationally used questionnaire with established validity and reliability.

Global clinical assessment of severity will be performed using the Clinical Dementia Rating Scale [53].

A trained neuropsychologist will test all patients. Together with neurologists and psychiatrists, the diagnosis of probable Alzheimer’s disease will be made according to the Diagnostic and Statistical Manual of Mental Disorders IV (Text Revision) and NINCDS–ADRDA criteria for Alzheimer’s disease [52]. The NINCDS–ADRDA criteria require a history of cognitive decline and evidence of impairment in memory and at least one other cognitive domain. Possible cases of Alzheimer’s disease (in NINCDS–ADRDA terminology) will also be included, i.e. persons who meet these criteria and also have another condition thought to be contributing to cognitive impairment will be included. To exclude a serious cerebral lesion, such as infection or a local lesion, all subjects will undergo magnetic resonance imaging within 6 months of the diagnosis.

For safety and to maintain oversight of the study, an electronic case report form provided by the Clinical Trial Centre of the University Hospital Zurich will be completed after each information, assessment and therapy session. The case report form contains checklists, test results, information about the patient (other therapies, medications, problems, etc.), adverse events, dates of assessments and the names of the investigators. The participants will be screened regularly for suicidality. In the case of an increased suicidality risk, even if not expected, the patients will be withdrawn from the study by the research team.

### Methods against biases

The proposed study employs several methods to avoid biases:

- *Selection bias* The randomization procedure is the gold standard to avoid this bias.
- *Detection bias* The baseline assessment is made before randomization to avoid this bias. In addition, the therapists will not collect any outcome data t post-test or follow-up assessments. This will be done by blinded members of the research team (single-blinded trial). Research assistants and therapists will be instructed during their training on the importance of maintaining the blinded status. The participants will be asked not to comment at post-test or follow-up assessments on the nature of their involvement in the study.
- *Performance bias (intervention fidelity)* To confirm that the intervention has been delivered and received as intended, a standardization procedure (manual, extensive training and session documentation, including an adherence checklist and supervision) has been established.
- *Attrition bias* Statistical analysis will include techniques to minimize a possible attrition bias, i.e., intention-to-treat analysis and imputing missing data. In the case of dropping out, it is intended to continue data collection whenever possible.

### Statistical analysis

Several analytical strategies are planned, to test the efficacy of the intervention. First, ANOVAs will be conducted to compare the mean outcome in depressive symptoms. The sex, age, caregiver’s relationship (spouse, child, close friend) and severity of cognitive impairment and depressive symptoms at baseline will be considered covariates. Second, individual growth curves will be analyzed, i.e., the rates and shapes of changes will be examined and individual growth curves will be compared, to investigate the differences. Third, multivariate analyses will be used to test the effect of the intervention with regard to the secondary and caregiver outcome measures. In this way, correlations between the outcome measures can be considered. Post-hoc analyses will be performed following a significant overall effect.

A complete dataset will be available if all four tests (pre-test, post-test and follow-ups 1 and 2) have been completed. Dropping out can occur if a participant (1) does not complete the pre-test, (2) decides, after information or randomization, against participation, or (3) starts with the treatment, but discontinues it. If fewer than 20 % of the items of one’s self-report instrument are missing, the missing values are imputed using the mean of the scale for this participant. If the whole scale is missing, the value will be imputed using the expectation-maximization algorithm.

The data and all appropriate documentation will be stored for a minimum of 10 years after the completion of the study, including the follow-up period.

### Regulatory issues

#### Ethical approval

Ethical approval has been obtained through the Swiss Ethics Committee in the Canton of Zurich (reference number 2009–0078/3). The study will be conducted in accordance with the principles enunciated in the current Declaration of Helsinki, the Guidelines of Good Clinical Practice issued by the International Conference on Harmonisation and the Swiss regulatory authority’s requirements. Participation in the study is voluntary. There are no negative consequences for non-participation or dropping out. Patients who decline to participate or drop out receive medical and psychosocial care as usual. The participants are informed of the study conditions in detail (verbally and in writing) and specific questions can be discussed. Ethical challenges may arise as a result of randomization. However, the control conditions are active treatment conditions, so participants in any study condition will benefit if they enrol in the study.

#### Consent

Consent to enter the study will be sought from each participant only after a full explanation has been given, an information leaflet offered and time allowed for consideration. Signatures to indicate participants’ consent will be obtained. The right of the participant to refuse to participate without giving reasons will be respected. After participants have entered the study, clinicians remain free to provide an alternative treatment to that specified in the protocol at any stage, if they consider it to be in the participant’s best interest, but the reasons for doing so will be recorded. In these cases, the participants remain within the study for the purposes of follow-up and data analysis. All participants are free to withdraw at any time from the protocol treatment without giving reasons and without prejudicing further treatment.

#### Confidentiality

The principal investigator will preserve the confidentiality of the participants in the study and is registered under the Data Protection Act.

#### Insurance

Insurance coverage will be provided by Zurich Versicherungsgesellschaft, Zurich, Switzerland (policy number 9.730.682), for a maximum of 3,000,000 Swiss francs.

#### Audits

The study may be subject to inspection and audit by regulatory bodies, to ensure adherence to the Guidelines of Good Clinical Practice, national law and regulatory requirements. The auditor will be independent from the investigators and the sponsor.

#### Adverse events

An adverse event is any untoward medical or psychological occurrence in a study subject, e.g., worsening of symptoms. A serious adverse event is any untoward medical or psychological occurrence or effect that results in death, is life-threatening (this refers to an event in which the subject was at risk of death at the time of the event but does not refer to an event which hypothetically might have caused death had it been more severe), requires hospitalization or prolongation of existing inpatients’ hospitalization, or results in persistent or significant disability or incapacity. Medical or psychological judgement will be exercised in deciding whether an adverse event is serious. Important adverse events that are not immediately life-threatening or do not result in death or hospitalization but may jeopardize the subject or may require intervention to prevent one of the other outcomes listed in the serious adverse event definition will also be considered serious.

All adverse events will be reported. Depending on the nature of the event, the following reporting procedures will be followed:

- *Non-serious adverse events* All such events, whether expected or not, will be recorded.
- *Serious adverse events* A serious adverse event form will be completed and faxed to the principal investigator within 24 hours. However, hospitalizations for an elective treatment of a pre-existing condition does not need to be reported as a serious adverse event.

The principal investigator will assess whether the event is:

- *‘related’*, i.e. results from the administration of any of the research procedures;
- *‘unexpected’*, i.e. is not listed in the protocol as an expected occurrence.

Reports of suspected related and unexpected serious adverse events will be submitted to the ethics committee within 15 days (or within 7 days for fatal or life-threatening serious adverse events) of an investigator becoming aware of the event.

## Discussion

This trial is a CBT-based, multicomponent treatment implemented with state-of-the-art randomized controlled trial methodology. A recent report on national dementia strategies concludes that any national dementia plan should include, among other things, the goals of improving early diagnosis and treatment, of improving support to the person with dementia living at home, as well as to the caregiver, and of improving training for healthcare professionals [86]. This trial has the potential to establish an empirically based psychological treatment for non-cognitive symptoms that reduce the quality of life of the person with dementia and the caregiver. The treatment manual will be published and training workshops will be offered, so that the information can be widely spread among healthcare professionals. This CBT-based treatment is potentially a side-effect-free alternative to medication for neuropsychiatric symptoms. In the case of affective and behavioural symptoms, there is a strong case for psychosocial intervention prior to resorting to medication [87]. Furthermore, health care costs might be reduced through decreased use of medication and delayed nursing home placement.

This treatment approach focuses not only on the person with dementia, but also on the caregiver and on the dyad. It will empower the caregiver to take part in the treatment and, at the same time, will improve the caregiver’s own mental health and well-being.

Treatment guidelines list a larger number of psychosocial interventions for dementia. However, empirical evidence for many of the approaches is lacking, as recent reviews conclude [9–11]. This trial, with state-of-the-art methodology, has the potential to provide evidence for future guidelines for the psychological treatment of people with dementia and their caregivers.

### Trial status

The trial is in the recruiting phase at the time of manuscript submission.

## Abbreviations

ANOVA: analysis of variance
CBT: cognitive behavioural therapy
CBTAC: Cognitive behavioural treatment for mild Alzheimer’s patients and their caregivers
CERAD: Consortium to Establish a Registry for Alzheimer’s Disease
CONSORT: Consolidated Standards of Reporting Trials
NINCDS–ADRDA: National Institute of Neurological and Communicative Disorders and Stroke/Alzheimer’s Disease and Related Disorders Association
